# Urinary Phthalate Metabolite Concentrations and Diabetes among Women in the National Health and Nutrition Examination Survey (NHANES) 2001–2008

**DOI:** 10.1289/ehp.1104717

**Published:** 2012-07-13

**Authors:** Tamarra James-Todd, Richard Stahlhut, John D. Meeker, Sheena-Gail Powell, Russ Hauser, Tianyi Huang, Janet Rich-Edwards

**Affiliations:** 1 Division of Women’s Health, Department of Medicine, Connors Center for Women’s Health and Gender Biology, Brigham and Women’s Hospital and Harvard Medical School, Boston, Massachusetts, USA; 2 Department of Obstetrics and Gynecology, University of Rochester School of Medicine and Dentistry, Rochester, New York, USA; 3 Department of Environmental Health Sciences, University of Michigan School of Public Health, Ann Arbor, Michigan, USA; 4 Department of Epidemiology, and; 5 Department of Environmental Health, Harvard School of Public Health, Boston, Massachusetts, USA

**Keywords:** diabetes, di(2-ethylhexyl) phthalate, monobenzyl phthalate, mono-(3-carboxypropyl) phthalate, monoethyl phthalate, mono-isobutyl phthalate, mono-*n*-butyl phthalate, women

## Abstract

Background: Previous studies have shown that women have higher urinary concentrations of several phthalate metabolites than do men, possibly because of a higher use of personal care products. Few studies have evaluated the association between phthalate metabolites, diabetes, and diabetes-related risk factors among women.

Objective: We explored the association between urinary phthalate metabolite concentrations and diabetes among women who participated in a cross-sectional study.

Methods: We used urinary concentrations of phthalate metabolites, analyzed by the Centers for Disease Control and Prevention, and self-reported diabetes of 2,350 women between 20 and 79 years of age who participated in the NHANES (2001–2008). We used multiple logistic regression to estimate odds ratios (ORs) and 95% confidence intervals (CIs) and adjusted for urinary creatinine, sociodemographic characteristics, dietary factors, and body size. A secondary analysis was conducted for women who did not have diabetes to evaluate the association between phthalate metabolite concentrations and fasting blood glucose (FBG), homeostasis model assessment–estimated insulin resistance, and glycosylated hemoglobin A1c.

Results: After adjusting for potential confounders, women with higher levels of mono-*n*-butyl phthalate (MnBP), mono-isobutyl phthalate (MiBP), monobenzyl phthalate (MBzP), mono-(3-carboxypropyl) phthalate (MCPP), and three di-(2-ethylhexyl) phthalate metabolites (ΣDEHP) had an increased odds of diabetes compared with women with the lowest levels of these phthalates. Women in the highest quartile for MBzP and MiBP had almost twice the odds of diabetes [OR = 1.96 (95% CI: 1.11, 3.47) and OR = 1.95 (95% CI: 0.99, 3.85), respectively] compared with women in the lowest quartile. Nonmonotonic, positive associations were found for MnBP and ΣDEHP, whereas MCPP appeared to have a threshold effect. Certain phthalate metabolites were positively associated with FBG and insulin resistance.

Discussion: Urinary levels of several phthalates were associated with prevalent diabetes. Future prospective studies are needed to further explore these associations to determine whether phthalate exposure can alter glucose metabolism and increase the risk of insulin resistance and diabetes.

Among women, the prevalence of diagnosed diabetes doubled from 1980 to 2010, with the age-adjusted prevalence increasing from 2.9% to 5.9% [Centers for Disease Control and Prevention (CDC) 2011a]. Although increased body mass index (BMI) is a major risk factor of diabetes (Colditz et al. 1995; Flegal et al. 2010; Mokdad et al. 2000), other factors are thought to be involved in the type 2 diabetes (T2DM) epidemic (Stahlhut et al. 2007; Svensson et al. 2011; Thayer et al 2012). In particular, certain types of environmental endocrine-disrupting chemicals (EDCs) have been implicated as having the ability to alter both adiposity and insulin resistance (Hatch et al. 2008; Latini et al. 2009; Newbold 2010; Stahlhut et al. 2007). Phthalates may be among those EDCs with the joint ability to impact adipogenesis and dysregulate glucose metabolism (Casals-Casas et al. 2008; Desvergne et al. 2009; Grun and Blumberg 2007).

Phthalates are a class of chemicals used in the manufacturing of a variety of products (Crinnion 2010; Hauser and Calafat 2005; Romero-Franco et al. 2011). These chemicals are often used as plasticizers or solvents in food packaging, cosmetics, perfumes, nail polishes, flooring, and other industrial products. For the past 50 years, phthalate production has increased (Baillie-Hamilton 2002). Phthalate exposure is nearly ubiquitous, with > 75% of the U.S. population having detectable urine concentrations of many phthalate metabolites (Hauser and Calafat 2005).

Recent studies have suggested that phthalates may disrupt metabolism and adipogenesis (Casals-Casas et al. 2008; Desvergne et al. 2009; Grun and Blumberg 2007). Specifically, phthalates can bind to peroxisome proliferator-activated receptors (PPAR) alpha and gamma, which regulate glucose metabolism and adipogenesis, respectively (Casals-Casas et al. 2008; Desvergne et al. 2009; Grun and Blumberg 2007). Dysregulation of glucose metabolism, possibly through increased insulin resistance, is a hallmark of T2DM.

In a cross-sectional study of a representative sample of U.S. men participating in NHANES, Stahlhut et al. (2007) showed that exposure to higher levels of phthalates was associated with increased waist circumference and insulin resistance—both risk factors for T2DM. In a cross-sectional study of Mexican women, Svensson et al. (2011) found that higher exposure of certain phthalates was associated with T2DM. Although the prevalence of diabetes is similar among men and women in the United States, differences in fat storage and hormonal profiles related to insulin resistance exist (Tsatsoulis et al. 2009). If certain environmental chemicals have the ability to alter adiposity and insulin resistance, then exposure to these chemicals could vary by sex. Women may be particularly vulnerable to metabolic disruption of phthalates, given their higher concentrations of certain urinary phthalate metabolites (Silva et al. 2004).

In this study, we analyzed data from women 20–79 years of age who participated in the National Health and Nutrition Examination Survey 2001–2008 (NHANES). We estimated the association of diabetes with concentrations of monoethyl phthalate (MEP), mono-*n*-butyl phthalate (MnBP), mono-isobutyl phthalate (MiBP), monobenzyl phthalate (MBzP), mono-(3-carboxypropyl) phthalate (MCPP), and the sum of three di(2-ethylhexyl) phthalate (DEHP) metabolites [mono-(2-ethylhexyl) phthalate (MEHP), mono-(2-ethyl-5-hydroxyhexyl) phthalate (MEHHP), and mono-(2-ethyl-5-oxohexyl) phthalate (MEOHP)]. To estimate the association between these phthalates and diabetes, we adjusted for sociodemographic characteristics and behavioral factors. Because diet is one of the main sources of exposure for certain phthalates, we adjusted for dietary factors, including total caloric and fat intake (Grun and Blumberg 2007; Hauser and Calafat 2005). We also adjusted for BMI and waist circumference, which could not be ruled out as potential intermediates or confounders given the cross-sectional study design. To determine whether phthalate exposure was associated with factors that likely precede the onset of diabetes, we conducted a secondary analysis to estimate associations between phthalate metabolites and fasting blood glucose (FBG), homeostasis model assessment–estimated insulin resistance (HOMA-IR), and glycosylated hemoglobin A1c (A1c) among women who did not have a diagnosis of diabetes. We hypothesized that higher urinary phthalate levels would be associated with an increased odds of prevalent diabetes.

## Methods

*Study population.* NHANES is a survey of a nationally representative sample of the non-institutionalized U.S. civilian population conducted by the CDC’s National Center for Health Statistics. NHANES uses a complex, multi-staged, stratified, clustered design; oversampling is conducted to ensure that certain subgroups are well represented, such as blacks, Mexican-Americans, and people with low incomes. Each year between 2001 and 2008, NHANES enrolled approximately 5,000 men and women. Data were collected on demographic, dietary, and behavioral factors using in-home questionnaires. Anthropometric information was collected through physical examinations conducted in mobile exam units. Written informed consent was obtained from all NHANES participants. Data on the NHANES web site are de-identified and publically accessible. Additional details on study procedures, data documentation, and questionnaires are available elsewhere (CDC 2011b).

*Phthalate metabolite measurements.* Phthalate metabolites were measured in a random, one-third subsample of NHANES participants with urine measurements. Urine samples were collected and frozen at –20°C. These samples were shipped to the CDC’s National Center for Environmental Health (Atlanta, GA) for analysis. Phthalate metabolites, rather than their parent compounds, were analyzed to reduce potential exposure misclassification (Latini 2005). The metabolites were measured using solid-phase extraction, high performance liquid chromatography, and tandem mass spectrometry. These methods have been described in detail elsewhere (CDC 2011c; Hauser and Calafat 2005; Silva et al. 2004).

The phthalates measured between 2001 and 2008 varied by year. We selected phthalates that were measured in all years with > 60% of sample concentrations above the limit of detection (LOD). Based on these criteria, we examined MEP, MnBP, MiBP, MBzP, and MCPP. Because of the high correlation between DEHP metabolites, we calculated the molar sum of the DEHP metabolites—MEHP, MEHHP, and MEOHP (ΣDEHP). Exposures to individual phthalate metabolites and ΣDEHP were classified according to quartiles based on the overall population phthalate levels, with the lowest quartile used as the referent category. For each phthalate, we evaluated outliers by their sampling weight (CDC 2011d; Thayer et al. 2012) and excluded those values that were substantially outside of range (> 99th percentile). Measured concentrations below the LOD were replaced with the LOD divided by the square root of two.

*Diabetes.* Participants who responded “yes” to the question “Other than during pregnancy, have you ever been told by a doctor or health professional that you have diabetes or sugar diabetes?” were classified as persons with diabetes. If respondents refused to answer the question, did not know the answer, or had a missing value, we excluded them from the analysis.

*FBG, HOMA-IR, and A1c.* FBG and fasting insulin levels were available for a subset of women who were randomly assigned to a morning fast, whereas A1c was available for the full sample. We calculated HOMA-IR as an indicator of insulin resistance: [blood glucose (millimoles per liter) × insulin levels (microunits per milliliter)/22.5].

*Covariates.* We considered the age, race/ethnicity, highest level of education, poverty status, fasting time, total caloric, and total fat intake of the participants as potential confounders. We also evaluated BMI and waist circumference as potential intermediates.

The age of the respondents was categorized as 20–29, 30–39, 40–49, 50–59, 60–69, or 70–79 years. We restricted our upper age limit to < 80 years, because participants ≥ 80 years of age were assigned an age of 80 or 85 in some surveys to protect confidentiality. Race/ethnicity was categorized as Mexican-American, black, white (reference), and other (including “other Hispanic” and mixed race). We categorized education as high school graduate or less (reference), some college, college graduate or higher. Poverty status was classified as poverty income ratio < 1 versus ≥ 1 (reference) based on the federal-based poverty threshold and household income information (Ali et al. 2011). Fasting time was modeled as a continuous variable. Physical activity was categorized as any self-reported vigorous or moderate physical recreation activity versus none (reference), and smoking status was categorized as current, past, or never (reference).

Total caloric intake and total fat intake were taken from the summary results of the 24-hr dietary history. From 2001 to 2002, dietary food records were collected for one day. From 2003 to 2008, a second day was also assessed, in which case we used the mean of the two values. We evaluated these variables in quartiles, with the lowest quartile as the reference group.

BMI was categorized as underweight (< 18.5 kg/m^2^), normal weight [18.5–24 kg/m^2^ (reference)], overweight (25–29 kg/m^2^), and obese (≥ 30 kg/m^2^). Waist circumference was dichotomized at the median [< 94.5 cm (reference) vs. ≥ 94.5 cm].

*Statistical analysis.* When evaluating the association between urinary phthalate metabolites and diabetes, our primary analysis took into account the complex sampling scheme of NHANES. We performed multivariable logistic regression using PROC SURVEYLOGISTIC. We constructed four separate models to estimate the association between each phthalate metabolite and diabetes. All models were adjusted for urinary creatinine as a covariate; this adjustment is an alternative to using creatinine-corrected phthalate levels (Barr et al. 2005). We considered a variable a confounder if its inclusion in the model changed the beta coefficient for any of the phthalate exposure quartiles by > 10%. We evaluated four main models: Model 1 represented our crude model, which adjusted for urinary creatinine only; model 2 adjusted for urine creatinine plus fasting time and sociodemographic factors (age, race/ethnicity, education, poverty status); model 3 adjusted for model 2 variables plus behavioral factors. For MnBP, MEP, and MBzP, behavioral factors included physical activity, smoking status, total caloric intake, total fat intake. For MCPP and MiBP, behavioral factors included only total caloric and fat intake, whereas for ΣDEHP these factors included total caloric and fat intake, as well as physical activity. Model 4 adjusted for model 3 variables, and included BMI and waist circumference. For the MiBP, MCPP, and ΣDEHP models, we did not include physical activity or smoking status, as these variables did not alter the beta for these phthalates by > 10%. We evaluated the association between urine creatinine (categorized into quartiles) and diabetes using logistic regression; we did not find urine creatinine to be significantly associated with diabetes in this analysis. Odds ratios (ORs) and 95% confidence intervals (CIs) were calculated to estimate the association between each phthalate and diabetes using appropriate sample weights, strata, and cluster variables for this subgroup analysis (CDC 2011e).

We conducted a secondary analysis for those women who did not self-report diabetes in our study population. Given the highly skewed distribution of FBG, HOMA-IR, and A1c values, even after log transformation, we used median regression to explore the association between phthalates and the three outcomes of interest (Burgette et al. 2011; SAS Institute Inc. 2011). We log-transformed HOMA-IR (Bonora et al. 2000; Emoto et al. 1999; Hermans et al. 1999). Exact 95% CIs were generated by bootstrap resampling with 5,000 repeats. For the secondary analysis that evaluated the association between urinary phthalate concentrations and FBG and HOMA-IR, we did not use sample weights, strata, or cluster variables, because this analysis was based on two somewhat overlapping subsets of the overall NHANES (2001–2008) study population (i.e., participants randomly selected for the urinary phthalate laboratory analysis and those randomly selected for a morning fasting blood draw). To maintain consistency with respect to model selection, we used models without sample weights, strata or cluster variables for the analysis of phthalate metabolite concentrations and hemoglobin A1c, as well. We adjusted for variables used in the creation of weights in our final models (Korn and Graubard 1991). All analyses were conducted using SAS software (version 9.2; SAS Institute Inc., Cary, NC).

## Results

*Descriptive statistics.* A total of 3,064 women between 20 and 79 years of age had available data on both diabetes and phthalates. For the present analysis, we excluded women who were pregnant (*n* = 294), had kidney problems (*n* = 62), or both (*n* = 2), and women with missing covariates included in our models (*n* = 356); a total of 2,350 women were included in our primary analysis. Because women with outlier information on phthalates were excluded, total *n* varied slightly for each phthalate metabolite (21–25 additional women were excluded). Analyses of associations with FBG, HOMA-IR, and A1c values included 985, 971, and 2,092 women, respectively. An additional 6–18 women were excluded from the secondary analysis due to outlying phthalate information.

Table 1 summarizes the weighted geometric means (with 95% CI) of each phthalate for the study population. Approximately 9% of women reported that a physician had diagnosed them with diabetes (*n* = 217). As expected, women with diabetes were significantly more likely to have a higher BMI and waist circumference, and to be older, non-white, and to have lower physical activity (all *p* < 0.05) (data not shown).

**Table 1:** Study population characteristics of women 20–79 years of age (NHANES 2001–2008).

Characteristic	n (%)	MEP	MnBP	MiBP	MBzP	MCPP	∑DEHP (× 100)
Total		2,350 (100)		164.8 (150.5, 180.3)		17.7 (16.6, 18.9)		3.7 (3.5, 3.9)		9.7 (9.0, 10.6)		2.0 (1.8, 2.1)		11.1 (10.3, 12.0)
Age (years)														
20–29		429 (18.3)		190.0 (161.3, 223.8)		23.9 (20.9, 27.3)		5.0 (4.4, 5.7)		16.4 (14.3, 18.9)		2.7 (2.3, 3.0)		16.6 (14.5, 19.0)
30–39		417 (17.7)		185.7 (156.5, 220.3)		19.0 (16.5, 21.8)		4.3 (3.7, 4.9)		11.9 (10.1, 13.9)		2.1 (1.8, 2.4)		12.9 (11.1, 14.9)
40–49		483 (20.6)		199.7 (176.1, 226.5)		16.9 (14.7, 19.6)		3.7 (3.3, 4.3)		9.5 (8.2, 11.1)		1.9 (1.7, 2.1)		10.2 (8.9, 11.7)
50–59		358 (15.2)		129.6 (106.7, 157.4)		16.2 (14.1, 18.7)		3.2 (2.8, 3.7)		7.4 (6.2, 8.9)		1.8 (1.6, 2.1)		9.6 (8.3, 11.2)
60–69		394 (16.8)		127.9 (102.9, 159.1)		13.4 (11.2, 16.2)		2.7 (2.2, 3.2)		5.9 (4.8, 7.2)		1.5 (1.3, 1.8)		8.5 (7.4, 9.8)
70–79		269 (11.5)		125.7 (105.6, 149.6)		15.6 (13.3, 18.3)		2.5 (2.2, 3.0)		7.3 (6.1, 8.7)		1.6 (1.4, 1.9)		8.1 (6.9, 9.5)
Race/ethnicity														
Mexican-American		469 (20.0)		248.9 (213.5, 290.2)		21.2 (18.3, 24.5)		5.3 (4.6, 6.1)		10.4 (8.6, 12.7)		2.1 (1.8, 2.5)		11.2 (9.8, 12.9)
White		1,142 (48.6)		142.0 (128.0, 157.6)		16.0 (14.8, 17.2)		3.1 (2.9, 3.4)		8.9 (8.1, 9.9)		1.9 (1.7, 2.0)		10.2 (9.3, 11.2)
Black		510 (21.7)		334.3 (293.3, 381.0)		27.6 (24.8, 30.8)		6.8 (6.1, 7.6)		14.8 (12.6, 17.4)		2.2 (2.0, 2.4)		17.3 (15.6, 19.1)
Other		229 (9.7)		159.1 (127.1, 199.2)		20.1 (16.5, 24.5)		4.9 (4.1, 5.8)		10.5 (7.9, 13.9)		2.1 (1.7, 2.6)		12.3 (9.9, 15.2)
Education														
≤ High school		1,146 (48.8)		188.1 (165.7, 213.4)		18.7 (17.1, 20.4)		3.8 (3.5, 4.2)		10.6 (9.7, 11.7)		2.0 (1.9, 2.2)		11.2 (10.1, 12.5)
Some college		736 (31.3)		176.9 (154.8, 202.1)		18.1 (15.9, 20.7)		3.7 (3.3, 4.2)		10.8 (9.3, 12.5)		2.0 (1.8, 2.3)		11.3 (9.9, 12.9)
≥ College graduate		468 (19.9)		120.9 (103.0, 142.0)		15.8 (14.1, 17.8)		3.4 (2.9, 3.9)		7.3 (6.3, 8.6)		1.8 (1.6, 2.1)		10.7 (9.4, 12.2)
Family poverty to income ratio														
Above poverty (≥ 1)		1,887 (80.3)		157.7 (143.6, 173.2)		16.8 (15.7, 18.1)		3.5 (3.2, 3.7)		9.0 (8.2, 9.8)		1.9 (1.8, 2.0)		10.8 (10.0, 11.7)
Below poverty (< 1)		463 (19.7)		215.1 (180.3, 256.5)		24.1 (20.5, 28.5)		5.1 (4.4, 6.0)		16.0 (13.4, 19.1)		2.4 (2.1, 2.8)		13.0 (10.7, 15.7)
Smoking status														
Never smoker		1,393 (59.3)		159.9 (144.6, 176.9)		16.9 (15.6, 18.4)		3.7 (3.4, 4.0)		9.6 (8.8, 10.5)		2.0 (1.8, 2.1)		11.5 (10.3, 12.8)
Current smoker		490 (20.9)		171.7 (148.4, 198.7)		21.5 (18.8, 24.5)		3.8 (3.3, 4.4)		11.7 (10.1, 13.4)		2.0 (1.8, 2.3)		11.3 (9.9, 13.0)
Past smoker		467 (19.9)		170.4 (143.9, 201.8)		16.3 (14.5, 18.3)		3.5 (3.0, 4.1)		8.3 (6.9, 10.0)		1.9 (1.7, 2.2)		9.9 (8.7, 11.4)
Moderate or vigorous physical activity														
Yes		1,343 (57.2)		154.9 (139.6, 171.9)		17.0 (15.7, 18.3)		3.5 (3.2, 3.7)		9.5 (8.5, 10.6)		2.0 (1.8, 2.1)		11.1 (10.3, 12.0)
No		1,007 (42.9)		184.8 (159.4, 214.1)		19.3 (17.2, 21.6)		4.1 (3.6, 4.6)		10.2 (9.0, 11.4)		2.0 (1.8, 2.1)		11.1 (9.8, 12.6)
Total calories (kcal)														
< 1,464		830 (35.3)		174.7 (154.0, 198.2)		18.9 (17.0, 20.9)		3.8 (3.4, 4.3)		10.1 (8.9, 11.4)		2.0 (1.8, 2.2)		10.4 (9.4, 11.5)
1,464 to < 1,942		700 (29.8)		165.5 (142.9, 191.7)		16.5 (14.7, 18.5)		3.4 (3.0, 3.8)		8.8 (7.8, 9.9)		1.8 (1.6, 2.0)		10.8 (9.6, 12.2)
1,942 to < 2,550		542 (23.1)		144.4 (124.8, 167.1)		16.1 (14.4, 18.0)		3.5 (3.1, 4.0)		9.2 (7.9, 10.8)		1.8 (1.7, 2.1)		11.0 (9.6, 12.5)
≥ 2,550		278 (11.8)		183.7 (149.6, 225.7)		22.0 (18.4, 26.4)		4.5 (3.7, 5.4)		12.7 (10.4, 15.7)		2.6 (2.2, 3.2)		14.6 (12.1, 17.6)
Total fat (g)														
< 49.3		756 (32.2)		172.6 (149.9, 198.8)		18.5 (16.4, 20.8)		3.9 (3.5, 4.3)		9.6 (8.5, 10.9)		1.9 (1.7, 2.1)		10.2 (9.2, 11.4)
49.3 to < 70.6		679 (28.9)		147.9 (126.0, 173.7)		15.9 (14.0, 18.1)		3.4 (3.0, 3.8)		8.7 (7.5, 10.2)		1.9 (1.7, 2.1)		10.1 (8.9, 11.4)
70.6 to < 97.5		561 (23.9)		163.8 (144.2, 186.0)		17.2 (15.7, 18.9)		3.5 (3.2, 3.9)		9.7 (8.5, 11.2)		1.9 (1.7, 2.2)		12.1 (10.7, 13.7)
≥ 97.5		354 (15.1)		185.8 (151.2, 228.4)		21.2 (18.0, 24.9)		4.2 (3.6, 4.9)		12.1 (9.9, 14.7)		2.4 (2.1, 2.8)		13.5 (11.8, 15.4)
BMI (kg/m2)														
Underweight (< 18.5)		48 (2.0)		112.3 (72.3, 174.3)		14.9 (10.2, 21.9)		2.9 (2.0, 4.3)		7.1 (5.1, 9.9)		1.6 (1.1, 2.2)		9.9 (7.2, 13.6)
Normal (18.5 to < 24)		702 (29.9)		134.9 (115.6, 157.4)		15.0 (13.4, 16.9)		3.1 (2.8, 3.4)		7.6 (6.5, 8.8)		1.7 (1.5, 1.9)		9.1 (8.1, 10.3)
Overweight (25 to < 30)		707 (30.1)		171.4 (149.6, 196.4)		18.7 (17.0, 20.5)		3.8 (3.4, 4.2)		10.1 (8.9, 11.4)		2.1 (1.9, 2.3)		11.3 (10.1, 12.6)
Obese (≥ 30)		893 (38.0)		202.3 (177.9, 230.0)		20.5 (18.7, 22.3)		4.4 (4.0, 4.9)		12.6 (11.4, 13.9)		2.2 (2.1, 2.4)		13.6 (12.5, 14.9)
Waist circumference (cm)														
< 94.5		1,194 (50.8)		151.7 (135.7, 169.7)		16.4 (15.1, 17.8)		3.4 (3.1, 3.7)		8.7 (7.8, 9.8)		1.8 (1.7, 2.0)		10.2 (9.2, 11.3)
≥ 94.5		1,156 (49.2)		182.9 (164.0, 204.0)		19.6 (17.9, 21.4)		4.1 (3.7, 4.5)		11.2 (10.1, 12.3)		2.1 (2.0, 2.3)		12.4 (11.3, 13.6)
Diabetes status														
Yes		215 (9.2)		188.2 (138.0, 256.6)		20.1 (17.1, 23.7)		4.5 (3.7, 5.5)		11.5 (9.5, 14.0)		2.2 (1.8, 2.7)		12.6 (10.6, 15.0)
No		2,135 (90.9)		163.3 (149.0, 178.9)		17.6 (16.4, 18.8)		3.6 (3.4, 3.9)		9.6 (8.8, 10.5)		1.9 (1.8, 2.1)		11.0 (10.2, 11.9)
Values are geometric mean (95% CI).														

*Association between phthalates and diabetes.* In Table 2, we present estimated associations between phthalate metabolites and diabetes. Statistically significant crude associations (model 1) were evident between MnBP, MiBP, MBzP, and ΣDEHP with diabetes prevalence. Although some associations appeared to have a nonmonotonic, increasing odds of diabetes, others seemed to suggest a threshold for an increasing odds of diabetes. Only MiBP appeared to have a monotonically increasing association with diabetes across quartiles for the crude associations, with the fourth quartile of MiBP conferring a 1.85 increased odds of diabetes (95% CI: 1.04, 3.27). On the other hand, there was evidence of a threshold for MBzP and MCPP, in which greater than median levels of these phthalates were associated with an increased odds of prevalent diabetes. MnBP and ΣDEHP had nonmonotonic associations with prevalent diabetes. In fact, the strongest association for these phthalates appeared to be the third exposure quartile. No apparent association existed between MEP and diabetes.

**Table 2:** Association [OR (95% CI)] between urinary phthalate metabolites and diabetes among women 20–79 years of age (NHANES 2001–2008).

Urinary phthalate metabolite	Model 1^a^	Model 2^b^	Model 3^c^	Model 4^d^
MEP							
Q1							
Q2	1.04 (0.65–1.68)		1.00 (0.63–1.59)		0.95 (0.60–1.51)		0.93 (0.58–1.49)
Q3	1.08 (0.62–1.88)		1.17 (0.64–2.13)		1.09 (0.61–1.96)		1.19 (0.65–2.20)
Q4	1.10 (0.58–2.06)		0.94 (0.49–1.80)		0.89 (0.47–1.67)		0.89 (0.48–1.68)
MnBP							
Q1							
Q2	1.37 (0.84–2.24)		1.32 (0.80–2.18)		1.29 (0.78–2.13)		1.31 (0.78–2.22)
Q3	2.01 (1.21–3.36)		1.76 (1.05–2.94)		1.71 (1.04–2.81)		1.73 (1.01–2.96)
Q4	1.32 (0.76–2.28)		1.06 (0.59–1.89)		1.06 (0.61–1.85)		1.14 (0.63–2.04)
MiBP							
Q1							
Q2	1.04 (0.66–1.66)		1.06 (0.67–1.68)		1.04 (0.66–1.67)		1.03 (0.64–1.67)
Q3	1.47 (0.85–2.53)		1.65 (0.91–2.98)		1.69 (0.93–3.06)		1.71 (0.92–3.16)
Q4	1.85 (1.04–3.27)		1.97 (0.99–3.93)		1.95 (0.99–3.85)		1.80 (0.89–3.65)
MBzP							
Q1							
Q2	0.81 (0.43–1.51)		0.85 (0.45–1.60)		0.78 (0.41–1.49)		0.84 (0.44–1.60)
Q3	1.73 (1.12–2.66)		1.84 (1.18–2.88)		1.80 (1.16–2.81)		1.90 (1.18–3.08)
Q4	1.60 (0.86–2.97)		1.95 (1.09–3.48)		1.96 (1.11–3.47)		1.99 (1.14–3.49)
MCPPe							
Q1							
Q2	0.78 (0.46–1.33)		0.85 (0.50–1.44)		0.83 (0.49–1.43)		0.76 (0.44–1.33)
Q3	1.46 (0.95–2.25)		1.54 (0.98–2.42)		1.55 (0.98–2.44)		1.47 (0.90–2.41)
Q4	1.45 (0.84–2.49)		1.62 (0.97–2.71)		1.68 (1.03–2.75)		1.64 (0.96–2.79)
∑DEHP (MEHP, MEHHP, and MEOHP)f							
Q1							
Q2	1.53 (0.92–2.54)		1.58 (0.97–2.57)		1.53 (0.95–2.48)		1.47 (0.90–2.40)
Q3	1.81 (1.10–2.99)		1.85 (1.13–3.02)		1.73 (1.03–2.91)		1.70 (0.96–3.03)
Q4	1.45 (0.84–2.51)		1.66 (0.90–3.05)		1.53 (0.82–2.87)		1.43 (0.75–2.75)
Q, quartile. For each of the metabolites, Q1 is the reference. aAdjusted for urine creatinine. bAdjusted for urine creatinine, age, race/ethnicity, education, poverty status, and fasting time. cAdjusted for urine creatinine, age, race/ethnicity, education, poverty status, fasting time, total caloric intake, total fat intake, smoking status, and physical activity. dAdjusted for urine creatinine, age, race/ethnicity, education, poverty status, fasting time, total caloric intake, total fat intake, smoking status, physical activity, BMI, and waist circumference. eSmoking and physical activity were not included in this analysis. fSmoking was not included in this analysis.							

Adjustments made in models 2 and 3 only slightly altered associations between all phthalates and prevalent diabetes. Having higher than median (i.e., third or fourth quartile levels of exposure) for MBzP and MCPP continued to confer an increased odds of diabetes. Women in the third quartile (Q3) and in the fourth quartile (Q4) of MBzP had 1.80 and 1.96 times the odds of prevalent diabetes, respectively (95% CI for Q3: 1.16, 2.81; 95% CI for Q4: 1.11, 3.47). Likewise, women in Q3 and Q4 for MCPP had a 1.55 and 1.68 increased odds of diabetes, respectively (95% I for Q3: 0.98, 2.44; 95% CI for Q4: 1.03, 2.75). MnBP and ΣDEHP continued to have nonmonotonic associations with Q3 showing a higher odds of diabetes (OR for Q3 = 1.71; 95% CI: 1.04, 2.81 and OR for Q3 = 1.73; 1.03, 2.91, respectively). The monotonically increasing association seen for MiBP remained, with increasing levels of MiBP conferring an increased odds of diabetes. However, this association had borderline statistical significance. Additional adjustment for BMI and waist circumference did little to the alter these associations.

*Association between phthalates, FBG, HOMA-IR, and hemoglobin A1c.* In Table 3, we present the association between phthalates and biomarkers of diabetes risk. We found consistent associations between MBzP and MiBP and FBG, after adjustment for sociodemographic, behavioral, and dietary factors (model 1). For MBzP, we found an inverse association with FBG, with the median blood glucose level in the highest quartile being 2.27 mg/dL lower than the median FBG for participants in the lowest quartile (95% CI: –4.76, 0.21). However, this association did not reach statistical significance. On the other hand, MiBP showed an increasing monotonic association with FBG. Women with the highest quartile of MiBP had a 5.86 mg/dL higher median FBG compared with women in the lowest quartile (95% CI: 3.55, 8.17). These patterns held even after adjusting for BMI and waist circumference. No apparent patterns were present for other urinary phthalate metabolites and FBG.

**Table 3:** Association [difference in median value (95% CI)] between urinary phthalate metabolites and FBG, ln-HOMA-IR, and A1c among women 20–79 years of age without self-reported diabetes (NHANES 2001–2008).^a^

FBG (mg/dL)			ln(HOMA-IR)			A1c (%)		
Phthalates	Model 1b	Model 2c	Model 1b	Model 2c	Model 1b	Model 2c
MEP		n = 979				n = 965				n = 2,074		
Q1												
Q2		0.95 (–0.94, 2.85)		1.10 (–0.83, 3.04)		0.06 (–0.10, 0.23)		0.03 (–0.09, 0.14)		0.01 (–0.04, 0.06)		–0.02 (–0.07, 0.02)
Q3		1.18 (–0.91, 3.27)		0.38 (–1.91, 2.67)		0.07 (–0.08, 0.23)		0.01 (–0.11, 0.14)		–0.02 (–0.07, 0.03)		–0.03 (–0.07, 0.02)
Q4		–0.03 (–2.16, 2.09)		–0.61 (–2.99, 1.78)		0.10 (–0.07, 0.26)		–0.04 (–0.17, 0.09)		–0.03 (–0.08, 0.02)		–0.05 (–0.10, 0.00)
MnBP		n = 985				n = 971				n = 2,092		
Q1		REF		REF		REF		REF		REF		REF
Q2		–0.35 (–2.07, 1.38)		–0.62 (–2.62, 1.38)		0.09 (–0.06, 0.25)		0.04 (–0.08, 0.16)		0.01 (–0.04, 0.06)		0.00 (–0.04, 0.04)
Q3		–0.19 (–2.22, 1.83)		0.19 (–2.05, 2.43)		0.09 (–0.06, 0.24)		0.11 (–0.01, 0.23)		–0.02 (–0.08, 0.03)		–0.03 (–0.08, 0.02)
Q4		–0.03 (–2.35, 2.30)		–0.05 (–2.47, 2.36)		0.14 (–0.04, 0.31)		0.10 (–0.04, 0.24)		–0.03 (–0.09, 0.02)		–0.02 (–0.07, 0.03)
MBzP		n = 985				n = 971				n = 2,092		
Q1												
Q2		0.00 (–1.70, 1.70)		0.77 (–1.11, 2.64)		0.09 (–0.07, 0.25)		–0.01 (–0.12, 0.11)		0.01 (–0.04, 0.06)		–0.01 (–0.05, 0.04)
Q3		–1.13 (–3.24, 0.98)		–1.08 (–3.34, 1.18)		0.13 (–0.02, 0.28)		0.06 (–0.07, 0.19)		0.00 (–0.05, 0.05)		–0.03 (–0.08, 0.01)
Q4		–2.27 (–4.76, 0.21)		–2.80 (–5.32, –0.28)		0.10 (–0.09, 0.29)		–0.07 (–0.22, 0.09)		–0.03 (–0.09, 0.03)		–0.03 (–0.09, 0.02)
MCPP		n = 985				n = 971				n = 2,092		
Q1												
Q2		1.06 (–0.90, 3.02)		0.98 (–1.20, 3.15)		0.04 (–0.13, 0.20)		0.01 (–0.11, 0.13)		–0.04 (–0.09, 0.00)		–0.04 (–0.09, 0.00)
Q3		0.65 (–1.42, 2.73)		0.01 (–2.23, 2.24)		0.02 (–0.14, 0.17)		–0.03 (–0.16, 0.10)		–0.02 (–0.07, 0.03)		–0.01 (–0.06, 0.04)
Q4		–0.06 (–2.24, 2.12)		–0.49 (–3.01, 2.04)		–0.07 (–0.23, 0.10)		–0.01 (–0.15, 0.13)		–0.06 (–0.12, –0.01)		–0.07 (–0.12, –0.01)
MiBP		n = 985				n = 971				n = 2,092		
Q1												
Q2		3.08 (1.22, 4.93)		3.03 (1.05, 5.00)		0.13 (–0.02, 0.28)		0.13 (0.01, 0.25)		0.03 (–0.01, 0.08)		0.03 (–0.01, 0.08)
Q3		3.50 (1.45, 5.54)		3.17 (1.17, 5.17)		0.08 (–0.08, 0.25)		0.10 (–0.01, 0.21)		0.03 (–0.02, 0.09)		0.04 (0.00, 0.09)
Q4		5.86 (3.55, 8.17)		6.04 (3.81, 8.28)		0.22 (0.06, 0.38)		0.18 (0.06, 0.31)		0.01 (–0.05, 0.07)		0.01 (–0.04, 0.07)
∑DEHP		n = 976				n = 962				n = 2,074		
Q1												
Q2		0.35 (–1.50, 2.19)		–0.13 (–2.08, 1.82)		0.12 (–0.04, 0.29)		0.01 (–0.11, 0.13)		0.04 (–0.01, 0.08)		0.02 (–0.02, 0.06)
Q3		–1.24 (–3.37, 0.89)		–1.75 (–3.93, 0.44)		0.10 (–0.04, 0.24)		0.05 (–0.07, 0.16)		–0.01 (–0.06, 0.04)		–0.03 (–0.07, 0.02)
Q4		0.25 (–1.94, 2.44)		0.01 (–2.34, 2.36)		0.19 (0.02, 0.35)		0.13 (0.01, 0.25)		0.01 (–0.05, 0.07)		–0.02 (–0.07, 0.03)
Q, quartile. For each of the phthalate categories, Q1 is the reference. aMedian regression used to conduct analysis. bAdjusted for urine creatinine, age, race/ethnicity, education, poverty status, fasting time, total caloric intake, total fat intake, smoking status, and physical activity. cAdjusted for urine creatinine, age, race/ethnicity, education, poverty status, fasting time, total caloric intake, total fat intake, smoking status, physical activity, BMI, and waist circumference.												

After adjusting for sociodemographic, behavioral, and dietary factors, we found an association between certain phthalate metabolites and HOMA-IR. Women with higher MiBP levels had higher median ln-HOMA-IR levels. This association was not linear, but the highest quartile conferred a higher median ln-HOMA-IR level compared with participants in the lowest quartile of MiBP (adjusted β for ln-HOMA-IR = 0.22; 95% CI: 0.06, 0.38). Likewise, DEHP had a nonmonotonic positive association with ln-HOMA-IR, with the highest quartile of MiBP conferring higher ln-HOMA-IR levels (adjusted β for ln-HOMA-IR = 0.19; 95% CI: 0.02, 0.35). Associations between other urinary phthalate metabolites and ln-HOMA-IR were not present. Furthermore, associations between urinary phthalate metabolites and hemoglobin A1c did not appear among this subset of women without diabetes. These associations were similar with further adjustment of BMI and waist circumference (model 2).

## Discussion

Women with higher urine levels of MnBP, MiBP, MBzP, MCPP, and ΣDEHP were more likely to have reported diabetes than were women with the lowest levels, even after accounting for sociodemographic, behavioral, and dietary factors. The addition of BMI attenuated some associations, whereas others became stronger. Nonmonotonic associations were seen for MnBP and ΣDEHP, with the greatest increased odds being seen among women in Q3. Although evidence of a threshold effect appeared to be present for MBzP and MCPP, increasing levels of MiBP were associated with an increasing odds of diabetes. The strongest associations were seen among women with high levels of MiBP and MBzP, who had almost twice the odds of prevalent diabetes as women with the lowest level of exposure. Among women without self-reported diabetes, MiBP was positively associated with FBG, and MiBP and ΣDEHP were positively associated with HOMA-IR. No associations were present for urinary phthalate metabolites and hemoglobin A1c levels.

Our findings for phthalate metabolites and prevalent diabetes are similar to Svensson et al. (2011) who reported that higher levels of the ΣDEHP metabolites MEHHP and MEOHP were associated with diabetes in a study of 221 Mexican women. In the present study, ΣDEHP was associated with insulin resistance among women who did not report a diagnosis of diabetes. Because insulin resistance often precedes T2DM, this finding suggests that DEHP might affect T2DM risk via effects on insulin resistance. In addition, we found other phthalate metabolites to be associated with an increased odds of diabetes, as well as FBG levels and insulin resistance. These findings suggest that other phthalates might affect T2DM risk either through glucose dysregulation or insulin resistance.

Phthalates could potentially impact diabetes risk in a number of ways. By binding to PPAR gamma, it has been posited that phthalates can up-regulate adipogenic genes leading to increased obesity in an environment where there is caloric excess (Grun and Blumberg 2007; Hurst and Waxman 2003). Grun and Blumberg postulate that coupling the up-regulation of adipocyte production with caloric excess could lead to metabolically inactive fat tissue (Grun and Blumberg 2007). This in turn could lead to insulin resistance along with metabolic dysregulation and an increased risk of diabetes (Grun and Blumberg 2007). Another potential pathway in which this could occur is the ability of phthalates to bind to PPAR alpha (Casals-Casas et al. 2008; Desvergne et al. 2009; Feige et al. 2010; Hurst and Waxman 2003; Lapinskas et al. 2005), which modifies lipid handling to control circulating glucose levels (Desvergne et al. 2009; Tsatsoulis et al. 2009). Although this pathway is less understood (Desvergne et al. 2009), if certain types of phthalates dysregulate glucose metabolism by impacting lipid handling, then these phthalates could impact diabetes risk by altering beta-cell insulin secretion (Tordjman et al. 2002).

A previous study positing metabolic effects of high phthalate metabolite concentrations found associations between phthalate metabolites and a measure of insulin resistance in male NHANES participants (Stahlhut et al. 2007). Animal and cellular models provide support for causal effects of phthalate exposures on diabetes risk. For example, rats given DEHP had altered insulin and glycogen levels, as well as increased blood glucose (Gayathri et al. 2004). *In vitro* models using liver cells have also found DEHP to reduce insulin receptor concentrations and glucose oxidation, suggesting that phthalates may lead to insulin resistance (Rengarajan et al. 2007). These previous findings could provide useful information about the present study’s findings of an association between higher levels of ΣDEHP and ln-HOMA-IR.

Other phthalates associated with diabetes did not appear to be associated with insulin resistance or FBG among women without diagnosed diabetes. In fact, MnBP was not significantly associated with FBG, HOMA-IR, or A1c. MBzP and MCPP were inversely associated with FBG and A1c, respectively, after controlling for BMI and waist circumference. These conflicting results could be due to residual confounding or to an artifact of the cross-sectional study design.

The present exploratory research study had a number of limitations. As a cross-sectional study, we cannot rule out the possibility of reverse causation. Phthalates are known to be in certain types of medications and medical devices, including medical products containing polyvinyl chloride used in intravenous bags and medical tubing. As such, it is possible that some of these associations are due to greater exposure to phthalates through increased use of certain medical devices and medications among women with diabetes (Hauser et al. 2004; Kelley et al. 2012). Future studies should longitudinally evaluate the association between phthalate levels and markers of insulin resistance and beta-cell functioning among nondiabetic women to better understand how phthalates could alter normal glucose metabolism and diabetes risk. Additionally, phthalate levels were measured using spot urines and do not account for temporal changes in exposure levels. Despite the rapid excretion of phthalates, which typically occurs within 24–48 hr (Hauser and Calafat 2005; Swan 2008) and vary within a person over time (Fromme et al. 2007), a number of studies show phthalate levels measured at one point to be modestly predictive of levels measured over the course of weeks or months (Hoppin et al. 2002; Peck et al 2010; Svensson et al. 2011). Although within-woman variability in phthalate levels is undoubtedly present, this type of measurement error would likely lead to nondifferential misclassification and a bias toward the null. Future studies should examine multiple urine samples taken over time to better estimate long-term phthalate exposure.

We were also unable to assess combinations of phthalates or adjust for exposure to different types of phthalates in this analysis. Exposure to a particular phthalate is unlikely to occur in isolation. Different phthalates may bind and activate different sets of genes, leading to potentially opposing effects on the normal functioning endocrine system. Correlation between phthalates and complexity of their interaction make it difficult to assess potential combinations of phthalates and their impact on human health outcomes, including diabetes. Future studies may need to assess this association, as high levels of various combinations of phthalates may increase the risk of diabetes.

Finally, type of diabetes was assessed by self-report and did not distinguish between type 1 and T2DM. Although we cannot differentiate diabetes type, we believe most (90–95%) of the persons had T2DM (Harris 1995). Also, self-reported diabetes may vary by education, age, and other factors. If more respondents of lower socioeconomic status inaccurately report not having diabetes, the resulting bias would be toward the null. Furthermore, a study using NHANES 2003–2006 reported that approximately 30% of diabetes cases were undiagnosed (Danaei et al. 2009), which could result in an underestimate of the true association between phthalate exposure and diabetes.

Despite the limitations of this study, there are many strengths. First, we explored this research question in a large study population of women who participated in NHANES over an 8-year period evaluating five individual phthalate metabolites and ΣDEHP metabolites. Second, our study population consisted of a representative sample of U.S. women. Third, we were able to control for several potential confounders, including sociodemographic, dietary, and behavioral factors. Fourth, to expand upon our findings of phthalates and prevalent diabetes, we conducted a secondary analysis among women without diagnosed diabetes. This analysis allowed us to explore the association between phthalates and markers of diabetes risk (FBG, HOMA-IR, and A1c). To our knowledge, this is the first study to examine the association between phthalates and diabetes in a large sample of women living in the United States.

In conclusion, urinary levels of MnBP, MiBP, MBzP, MCPP, and ΣDEHP were associated with diabetes among women. For women without diagnosed diabetes, some phthalate metabolites were positively associated with FBG and HOMA-IR. These findings suggest the need to further explore the association between phthalates, insulin resistance, and diabetes. If future studies determine causal links between phthalates and diabetes, then reducing phthalate exposure could decrease the risk of diabetes among women.
